# Current Trends in the Realm of Baking: When Indulgent Consumers Demand Healthy Sustainable Foods

**DOI:** 10.3390/foods8100518

**Published:** 2019-10-21

**Authors:** Mario M. Martinez, Manuel Gomez

**Affiliations:** 1 School of Engineering, University of Guelph, Guelph, ON N1G 2W1, Canada; 2 Food Technology Area, College of Agricultural Engineering, University of Valladolid, 34004 Palencia, Spain

The term “baked goods” encompasses multiple food products made from flour (typically wheat flour). Among them, bread has stood as a foundation in different cultures by providing energy, mostly from its starch fraction, while being low in fats and sugars. Nevertheless, breadcrumbs are categorized as having a high amount of rapidly digestible starch that has been associated with poor health outcomes, including type 2 diabetes, obesity, and cardiovascular disease, as well as other metabolic-related health problems. In this regard, the enrichment of bread with resistant starch (RS) ingredients is gaining prominence and can be definitively positioned as an impactful strategy to improve human health through diet. In this Special Issue, structural factors for the resistance to digestion and hydrothermal processing of clean label RS ingredients are reviewed by Roman et al. [1], who expanded the definition of each RS subtype to account for recently reported novel and natural non-digestible structures. The term baked goods also include cakes and cookies, which are rich in fats and sugars but represent an excellent choice for indulgent consumption. While bread may be an excellent food carrier of added nutritional and extranutritional compounds, such as proteins, dietary fibers and bioactive phytochemicals, the effort to improve the nutritional properties of cakes and cookies has focused on the elimination or reduction of fats and sugars associated with poor health outcomes. As an example, milk fats have typically been used in cake- and cookie-making, and their high content in calories and saturated fatty acids has encouraged food researchers and technologists to develop fat mimetics, as discussed in this Special Issue in the review by Huang et al. [2].

Many research groups have focused on the enrichment of baked goods with other plant-based ingredients of high nutritional value. Legume flours possess a high content of proteins with an amino acid profile complementary to that of cereals. As a result, the enrichment of breads with these flours has received significant attention over the last years, as revised in this Special Issue by Bresciani and Martí [3]. However, the incorporation of legume flours into baked goods usually results in lower organoleptic quality and the recipe must be re-adjusted to minimize these detrimental effects, as reported by Cunha et al. [4]. The use of ingredients from oil seeds is also becoming paramount in many recipes over the last years because they possess higher protein content than cereals and are rich in fiber, omega-6 and omega-3 essential fatty acids, and natural antioxidant compounds, including tocopherol, beta-carotene chlorogenic acid, caffeic acid and flavonoids. As discussed in the review written by De Lamo and Gómez [5], oil seeds can be added directly as whole seeds or as milled flour. In this Special Issue, Grasso et al. [6] considered the enrichment of cookies with defatted sunflower seed flour and Codina et al. [7] investigated the use of flaxseed flour in bread-making. As observed in these works, the nutritional improvement of baked goods derived from the use of the aforementioned nutrient-dense ingredients almost always worsens their physical quality. This may result in a critical loss of consumers’ acceptance and, therefore, the unfeasible translation of nutrient-dense ingredient incorporation to the commercial reality. This aspect is approached by Mellette et al. [8] using cakes made with whole flour. In this regard, Belorio et al. [9] found that optimization of the physical properties of a flour, specifically in terms of particle size, dramatically impacted the physical qualities of their baked good: cookies. In their work, the authors encourage ingredient technologists to optimize clean and simple technologies, such as milling mechanical fractionation, to produce clean label flours with optimum physical properties and successful commercial applications. 

Baked goods are also characterized as having a low protein content, although their high consumption makes them account for a significant fraction of the total recommended protein uptake. Nonetheless, protein scores in cereals, which are commonly the main ingredients in baked goods, are usually low due to a suboptimal amino acid profile and low protein digestibility. Interestingly, the overall protein digestibility is not only dependent on the protein source, but also the food processing methodology. The review written by Joye [10] provides an in-depth evaluation of protein digestibility as affected by the typical unit operations carried out during the manufacture of baked goods. 

Last but not least, this Special Issue considers the consumers’ increased awareness of the environment and sustainable food systems. In this regard, novel processing and breeding technologies have been reported as key contributors to reduced food waste and loss. As an example, the use of perennial grains has been reported to result in more efficient use of water, fertilizers, and soil nutrients, although their incorporation into foods is only possible if the quality of their resultant flours matches the expectations of both manufacturers and consumers. In this Special Issue, the impact of milling and tempering on the perennial grain intermediate wheatgrass was studied by Tyl et al. [11]. 

The works included in this Special Issue highlight the importance of holistically considering the nutritional improvement of baked goods by using sustainable plant-based ingredients and the optimization of the physical properties of such ingredients to result in successful commercial applications. However, scientists and technologists within the realm of baking should invest in translational research that provides a detailed understanding of food and food ingredient nano- and micro-structures, as well as the impact of processing and the development of successful recipes. 
